# Other PHAC publications

**DOI:** 10.24095/hpcdp.45.5.05

**Published:** 2025-05

**Researchers from the Public Health Agency of Canada also contribute to work published in other journals and books. Look for the following articles published in 2024 and 2025:**

Cohen A, **Lang JJ, Prince SA**, Colley RC, Tremblay MS, Chaput JP. Are adolescents who do physical activity with their parents more active and mentally healthier? Health Rep. 2025;36(1):19-33. https://www.doi.org/10.25318/82-003-x202500100002-eng

**Doan N, Srugo SA, Prince SA**, Colley RC, Rainham DG, Manyanga T, **Butler GP**, […] **Lang JJ**. Independent and joint associations of neighbourhood greenness and walkability with transportational and recreational physical activity among youth and adults in Canada. Prev Med Rep. 2025;50:102974. https://doi.org/10.1016/j.pmedr.2025.102974

Fortier J, Salmon S, Taillieu T, Stewart-Tufescu A, Macmillan HL, **Tonmyr L**, et al. Parents’ COVID-19 stressors and associations with self-rated health, symptoms of mental health problems, and substance use: a cross-sectional study. Facets. 2025;10:1-13. https://doi.org/10.1139/facets-2023-016

McIsaac DI, Kidd G, Gillis C, Branje K, Al-Bayati M, Baxi A, […], **Boland L**, et al. Relative efficacy of prehabilitation interventions and their components: systematic review with network and component network meta-analyses of randomised controlled trials. BMJ. 2025;388:e081164. https://doi.org/10.1136/bmj-2024-081164

**McKinnon B, Jahan R, Mazza J**. Social inequalities in youth mental health in Canada, 2007–2022: a population-based repeated cross-sectional study. Soc Psychiatry Psychiatr Epidemiol. 2025. https://doi.org/10.1101/2024.05.03.24306830

O’Neill CD, **Prince SA**, Mitra M, Reed JL. Considerations for assessing physical activity in those at risk of and living with cardiovascular disease. Can J Cardiol. 2025;41(3):553-7. https://doi.org/10.1016/j.cjca.2024.12.011

**Plebon-Huff S, Haji-Mohamed H, Gardiner H, Ghanem S, Koh J, LeBlanc AG**. Contextualization of diabetes: a review of reviews from Organisation for Economic Co-operation and Development (OECD) countries. Curr Diab Rep. 2025;25(1):19. https://doi.org/10.1007/s11892-024-01574-y

Singh B, Cadenas-Sanchez C, da Costa BG, Castro-Piero J, Chaput JP, Cuenca-Garca M, […] **Lang JJ**, et al. Comparison of objectively measured and estimated cardiorespiratory fitness to predict all-cause and cardiovascular disease mortality in adults: a systematic review and meta-analysis of 42 studies representing 35 cohorts and 3.8 million observations. J Sport Health Sci. 2024;14:100986. https://doi.org/10.1016/j.jshs.2024.100986

Veroniki AA, Hutton B, **Stevens A**, McKenzie JE, Page MJ, Moher D, et al. Update to the PRISMA guidelines for network meta-analyses and scoping reviews and development of guidelines for rapid reviews: a scoping review protocol. JBI Evid Synth. 2025;23(3):517-26. https://doi.org/10.11124/JBIES-24-00308

Weiler HA, Cooper M, Bertinato J, Hopperton KE, McCrea J, Rana H, […] **Nicholson C**, et al. Adherence to introduction of iron-rich solid foods recommendations for infants: an analysis of Canadian Community Health Survey data using cycles 2015–2018. J Nutr. 2024:S0022-3166(24)01253-7. https://doi.org/10.1016/j.tjnut.2024.12.026

Yin CY, Talarico R, **Scott MM**, Hakimjavadi R, Kierulf J, Webber C, et al. Development of a predictive model for loss of functional and cognitive abilities in long-term care home residents: a protocol. BMJ Open. 2025;15(1):e086935. https://doi.org/10.1136/bmjopen-2024-086935

